# Detection and classification of brain tumor using hybrid deep learning models

**DOI:** 10.1038/s41598-023-50505-6

**Published:** 2023-12-27

**Authors:** Baiju Babu Vimala, Saravanan Srinivasan, Sandeep Kumar Mathivanan, Mahalakshmi, Prabhu Jayagopal, Gemmachis Teshite Dalu

**Affiliations:** 1 School of Computer Science and Engineering, Vellore Institute of Technology, Vellore, 632014 Tamil Nadu India; 2 Department of Computer Science and Engineering, Vel Tech Rangarajan Dr. Sagunthala R&D Institute of Science and Technology, Avadi, Chennai, 600062 India; 3 School of Computing Science and Engineering, Galgotias University, Greater Noida, 203201 India; 4 Department of Mathematics, School of Applied Sciences, REVA University, Bangalore, Karnataka India; 5 School of Computer Science Engineering and Information Systems, Vellore Institute of Technology, Vellore, 632014 Tamil Nadu India; 6 Department of Software Engineering, College of Computing and Informatics, Haramaya University, POB 138, Dire Dawa, Ethiopia

**Keywords:** Health care, Medical research, Neurology, Computational neuroscience, Development of the nervous system, Diseases of the nervous system

## Abstract

Accurately classifying brain tumor types is critical for timely diagnosis and potentially saving lives. Magnetic Resonance Imaging (MRI) is a widely used non-invasive method for obtaining high-contrast grayscale brain images, primarily for tumor diagnosis. The application of Convolutional Neural Networks (CNNs) in deep learning has revolutionized diagnostic systems, leading to significant advancements in medical imaging interpretation. In this study, we employ a transfer learning-based fine-tuning approach using EfficientNets to classify brain tumors into three categories: glioma, meningioma, and pituitary tumors. We utilize the publicly accessible CE-MRI Figshare dataset to fine-tune five pre-trained models from the EfficientNets family, ranging from EfficientNetB0 to EfficientNetB4. Our approach involves a two-step process to refine the pre-trained EfficientNet model. First, we initialize the model with weights from the ImageNet dataset. Then, we add additional layers, including top layers and a fully connected layer, to enable tumor classification. We conduct various tests to assess the robustness of our fine-tuned EfficientNets in comparison to other pre-trained models. Additionally, we analyze the impact of data augmentation on the model's test accuracy. To gain insights into the model's decision-making, we employ Grad-CAM visualization to examine the attention maps generated by the most optimal model, effectively highlighting tumor locations within brain images. Our results reveal that using EfficientNetB2 as the underlying framework yields significant performance improvements. Specifically, the overall test accuracy, precision, recall, and F1-score were found to be 99.06%, 98.73%, 99.13%, and 98.79%, respectively.

## Introduction

Cancer is a medical condition characterized by the abnormal and uncontrolled growth of cells throughout the body. There are over 200 different types of cancer, such as lymphoma, blood cancer, breast cancer, skin cancer, and lung cancer. According to the World Health Organization (WHO), in 2018, approximately 9.6 million people worldwide lost their lives due to cancer^1^. Brain tumors are a prevalent and severe ailment, leading to reduced life expectancy in individuals of all age groups and genders. They are marked by the presence of an abnormal mass of brain cells, often arising from the sudden and irregular growth of brain tissues. The impact of brain tumors on an individual's life expectancy can be significant, dependent on factors like tumor type, size, location, and the availability of effective treatments. Effectively combating cancer hinges on being well-informed about risk factors and early detection methods^2^. Embracing a healthy lifestyle, undergoing regular medical check-ups, and following recommended screening guidelines can play a crucial role in reducing the cancer burden and improving overall health outcomes. It's always advisable to consult healthcare professionals for personalized information and guidance on cancer prevention and management^3^. Abnormal cells in the body don't remain stable in number; they multiply rapidly and spread, leading to the formation of tumors. A brain tumor can be categorized as either benign (noncancerous) or malignant (cancerous)^4^. When malignant tumors spread swiftly to other brain tissues, the patient's condition can deteriorate. The brain naturally replaces aging or damaged healthy cells with new ones. However, if old and damaged cells persist without timely removal, a tumor can develop, posing a serious concern. Early identification and treatment of a tumor are essential as it typically spreads to other tissues, increasing the chances of effective therapy and survival. Medical imaging plays a vital role in detecting brain tumors^5^. Both Magnetic Resonance Imaging (MRI) and Computed Tomography (CT) scans are valuable tools for providing crucial information about the presence and extent of abnormal brain tissues, facilitating timely follow-up procedures and enhancing our understanding of the tumor's condition. MRI is preferred over CT scans because it employs harmless radiation to generate images of internal body structures, in contrast to CT scans, which involve the use of damaging radiation^6^. MRI utilizes radio waves and a powerful magnetic field to offer precise details about soft tissues and internal anatomy within the human body. It produces various types of images, including FLAIR, T1, T1 contrast-enhanced, Proton Density (PD), and T2-weighted high-contrast grayscale images. These images are instrumental in classifying and distinguishing brain tumors based on their size, shape, and location^7^. Brain tumors are broadly categorized into two types: primary and secondary. Primary tumors originate within the brain cells, while secondary tumors spread to the brain from other parts of the body. The most common type of benign primary brain tumor is meningioma. On the other hand, gliomas make up 78% of malignant tumors and represent the most prevalent type of adult brain tumor^8^. The World Health Organization (WHO) has classified various tumor types into different groups based on their growth rate, malignancy ratio, recurrence, and severity, spanning from grade 1 to grade IV. Grades I and II are considered low-grade tumors, whereas III and IV are categorized as high-grade tumors. Classical Machine Learning techniques and algorithms have found extensive application in Computer-Aided Diagnostic (CAD) systems for classification tasks. These algorithms heavily rely on handcrafted features, which are then used as input for classifiers like Support Vector Machine (SVM), Decision Tree (DT), or k-Nearest Neighbor (KNN) for classification^9^.

Effective feature engineering requires domain knowledge and can be time-consuming, especially when dealing with large datasets. Moreover, manual feature engineering is prone to errors, which is a significant concern in the context of medical data, where accuracy is paramount and efficiency is desirable^10^. Deep Learning (DL) has demonstrated remarkable success in various computer vision applications, particularly with the convolutional neural network (CNN) architecture. In the realm of medical image processing, CNNs have also proven to be effective and efficient. CNNs automatically extract meaningful features from images, eliminating the need for manual feature engineering common in traditional machine learning methods^11^. The CNN architecture consists of several key components, including an input layer, convolutional layers, pooling layers, fully connected layers, and an output layer responsible for determining the class of the input image. The convolutional and pooling layers receive image input from the input layer, automatically extracting features and down-sampling feature maps during the process^12^. However, it's important to note that CNNs have limitations. They are data-hungry, requiring a large number of training samples to achieve higher accuracy and performance. Conversely, a lack of data samples may lead to reduced accuracy, which is particularly challenging in medical imaging where obtaining a substantial amount of labeled data can be difficult^13^. Additionally, CNNs come with a higher processing cost compared to traditional machine learning methods. Training CNNs demands powerful GPUs and ample random-access memory (RAM)^14^. To address these limitations, transfer learning is a powerful technique. It overcomes the challenges of CNNs by achieving good results with fewer training samples and reduced training time. This study focuses on applying transfer learning to classify brain tumors into three types: glioma, meningioma, and pituitary tumors. We utilize pre-trained EfficientNets as the foundation for transfer learning, specifically five variants, ranging from EfficientNetB0 to EfficientNetB4. These pre-trained models are fine-tuned using a CE-MRI brain tumor dataset to extract features and perform classification. Additionally, we incorporate several top layers into the pre-trained EfficientNets before training and evaluating them. In contrast to earlier deep CNN architectures, where scaling often involved arbitrary increases in width, height/depth, and resolution, we adopt transfer learning to enhance the model's generalizability for brain tumor classification. EfficientNets employ a uniform compound scaling strategy to systematically upscale the CNN architecture using predetermined sets of scaling coefficients. This approach makes EfficientNets a preferred choice over other pre-trained deep CNN designs due to their lightweight nature, computational efficiency, fewer network parameters, and superior performance on ImageNet. Additionally, EfficientNets require the fewest Floating-Point Operations Per Second (FLOPS) during inference. In this study, we conduct a series of tests to compare the robustness of our fine-tuned EfficientNets with other pre-trained models. We also explore the impact of data augmentation on the model's test accuracy. Finally, we present Grad-CAM visualization, which generates attention maps from the best model, effectively highlighting the tumor regions within brain images^15^.

The organization of this research contributions are as follows:

- Fine-tuning of pretrained EfficientNets is utilized as a transfer learning-based deep learning strategy for multiclass classification of brain tumor types.
- The convolutional base of five pre-trained EfficientNets variants, including EfficientNetB0 to EfficientNetB4, is fine-tuned using a publicly accessible CE-MRI dataset to categorize brain tumors into glioma, meningioma, and pituitary tumor.
- Extensive studies are conducted with updated variations of EfficientNets, namely EfficientNetB0 to EfficientNetB4, under various experimental scenarios. The Grad-CAM visualization of attention-maps is applied to brain tumor MRI sequences using a modified EfficientNetB2 model. This technique produces a coarse localization heatmap that highlights the tumorous area of the brain cells.
- The suggested fine-tuned EfficientNetB2 model demonstrates computational efficiency and successful generalization on previously encountered test examples. In terms of model accuracy, precision, sensitivity/recall, specificity, and F1 score, the proposed approach of fine-tuning modified EfficientNetB2 outperforms state-of-the-art methods.

The remaining chapters of the study are organized as follows: “Related work” presents a literature overview of the various cutting-edge approaches used in this work. In “Materials and methods”, the suggested technique is fully discussed. “Experimental setup” covers the dataset, system and software requirements, hyper-parameter settings, and assessment measures. The obtained results and their analysis are presented in “Experimental results and discussion”. “Conclusion” describes the performance analysis of the suggested method and compares it with state-of-the-art approaches. Finally, “Future work” concludes the study with a summary and future directions.

## Related work

This study aims to enhance the accuracy and efficiency of MRI scanners in categorizing brain tumors and identifying their types using AI algorithms, specifically Convolutional Neural Networks (CNN) and Deep Learning techniques. We employed five pre-trained models, namely Xception, ResNet50, InceptionV3, VGG16, and MobileNet, to train our brain tumor dataset. The F1-scores for these models on unseen images were as follows: Xception (98.75%), ResNet50 (98.50%), InceptionV3 (98.00%), VGG16 (97.50%), and MobileNet (97.25%). These high precision scores contribute to the early diagnosis of tumors, helping prevent physical adverse effects such as paralysis and other health problems^16^. Author introduces two significant techniques. The first is a modification to a genetic algorithm, termed Metaheuristics-based Genetic Algorithm (MGA), and the second is a technique known as Entropy-Kurtosis-based High Feature Values (EKbHFV). A novel threshold function further refines the characteristics of the GA. Subsequently, we utilize a multiclass Support Vector Machine (SVM) cubic classifier to combine the features derived from EKbHFV and MGA. In the experimental procedure, we achieve an accuracy of over 95% using two datasets, BRATS2018 and BRATS2019, without augmentation^17^. CNNs are expected to play a crucial role in future brain tumor research, as they are compatible with various image classification tasks, including recognition, localization, segmentation, registration, and detection. Segmentation allows us to extract various features from images by dividing them into distinct areas based on shared characteristics. Thanks to this study, brain tumors can now be classified with greater precision. According to experimental results, the developed model achieves an impressive accuracy rate of 95.71% in tumor detection on a publicly available dataset^18^.

The proposed procedure consists of five main stages. Firstly, we implement linear contrast stretching using edge-based histogram equalization and the discrete cosine transform (DCT). In the second stage, we extract features through deep learning. This involves the use of two pre-trained convolutional neural network (CNN) models, namely VGG16 and VGG19, achieved through transfer learning. The third stage combines the extreme learning machine (ELM) with a correntropy-based joint learning strategy for feature selection. In the fourth stage, robust covariant characteristics are merged into a single matrix using partial least squares (PLS). Finally, ELM is employed to classify the combined matrix. This technique was tested on the BraTS datasets, resulting in an accuracy of 97.8% for BraTs2015, 96.9% for BraTs2017, and 92.5% for BraTs2018^19^.

The author presents a classification method that utilizes a pre-trained GoogLeNet to extract features from brain MRI scans, taking advantage of deep transfer learning. These extracted features are then combined with well-established classifier models. The MRI dataset is sourced from Figshare, and the experiment employs a five-fold cross-validation procedure at the patient level. The proposed approach significantly outperforms existing methods, achieving a mean classification accuracy of 98%. The evaluation includes various metrics such as area under the curve (AUC), precision, recall, F-score, and specificity. Additionally, the article addresses the challenge of limited training samples by demonstrating effective system evaluation with fewer samples. The results emphasize the effectiveness of transfer learning in scenarios with limited available medical imagery, and the paper's analytical discussion explores misclassification cases^20^. The framework introduced by the author utilizes several pre-trained deep convolutional neural networks to extract intricate features from brain MR images. These extracted features are evaluated by diverse machine learning classifiers. To make predictions, the author selects the top three deep features with the best performance across multiple machine learning classifiers and combines them into a feature ensemble. The study uses three openly accessible brain MRI datasets to compare the effectiveness of different pre-trained models as deep feature extractors, various machine learning classifiers, and an ensemble of deep features for brain tumor classification^21^. The author applied a well-established deep learning architecture, the Convolutional Neural Network (CNN), to classify 3064 T1 weighted contrast-enhanced MR images of the brain into three distinct categories: gliomas, meningiomas, and pituitary tumors. The results of the proposed CNN classifier are impressive, achieving a high accuracy of 98.93% and a sensitivity of 98.18% for cropped lesions, an outstanding 99% accuracy and a sensitivity of 98.52% for uncropped lesions, and a commendable 97.62% accuracy and a sensitivity of 97.40% for segmented lesion images^22^. In this study, researchers assessed the performance of three distinct convolutional neural network architectures, namely AlexNet, GoogLeNet, and VGGNet, for the classification of brain tumors, including glioma, pituitary tumors, and meningioma. They used the MRI brain tumor dataset available on Figshare and explored various transfer learning approaches, including both fine-tuning and freeze methods. To improve the dataset's size, mitigate the risk of overfitting, and enhance the generalization of results, they applied data augmentation techniques to the MRI slices. The results were particularly impressive when using the fine-tuned VGG16 architecture, achieving classification and detection accuracy levels of up to 98.69%^23^. The author introduced a multi-grade brain tumor classification system based on convolutional neural networks (CNNs). Initially, they employed a deep learning approach to identify tumor locations within an MR image. To address the challenge of limited data, the utilization of MRI data for multi-grade brain tumor classification involved extensive data augmentation, which is crucial for effectively training the proposed system. Finally, additional data was used to refine a pre-trained CNN model for grading brain tumors. Experimental assessments conducted on both augmented and original data showcased the system's strong performance when compared to existing leading methods^24^.

The author proposed a neural model capable of analyzing sagittal, coronal, and axial MRI images from patients diagnosed with meningioma, glioma, and pituitary tumors without the need for prior preprocessing to remove elements like skull or vertebral column structures. To validate this approach, a comparison was conducted against conventional machine learning and deep learning techniques. The evaluation was carried out on a publicly available MRI imaging dataset, which included 3064 slices from 233 patients. Notably, this methodology outperformed other methods using the same dataset, achieving an impressive tumor classification accuracy of 0.973^25^. The proposed model underwent evaluation on a publicly available research dataset and yielded impressive results, including an F1-score of 99.66%, precision of 99.61%, recall of 100%, and accuracy of 99.67%. In comparison to various models like AlexNet, ResNet50, Darknet53, ShuffleNet, GoogLeNet, SqueezeNet, ResNet101, ExceptionNet, and MobileNetV2, this approach demonstrated the highest accuracy. In the realm of brain tumor classification in MRI images, the proposed model emerged as the most effective^26^. The study aimed to ease the burden on doctors by focusing solely on cranial MR scans indicating the presence of masses. Through empirical trials, an impressive accuracy rate of 97.18% was achieved in categorizing cranial MR images. The performance of this proposed technique surpassed that of other contemporary research efforts, as highlighted by the evaluation results. The experimental outcomes further validated the effectiveness of the proposed approach in facilitating computer-assisted brain tumor diagnosis^27^. The author introduced a robotic approach for the analysis of MRI scans to detect malignant brain tumors. Initially, a preprocessing phase was employed to enhance the quality of brain MRI scan images. Two pre-trained deep learning models were crucial for extracting meaningful features from the images. To consolidate the feature vectors obtained, the methodology involved applying partial least squares (PLS) to create a hybrid feature vector. Subsequently, agglomerative clustering was used to identify regions with the highest tumor concentration. Once resized and aligned, these insights were relayed to the central network for further classification. This approach significantly increased classification accuracy to 98.95%, outperforming current leading methods^28^. The proposed framework is trained and assessed using a dataset comprising 15,320 MRIs, with the results highlighting the superior performance of the fused feature vector over individual vectors in terms of classification accuracy. This method outperforms prevailing algorithms and elevates the classification accuracy to 99.7%. As a result, it is well-suited for application in clinical contexts, enabling the identification and categorization of brain tumors using magnetic resonance images^29^.

## Materials and methods

**Figure 1:**
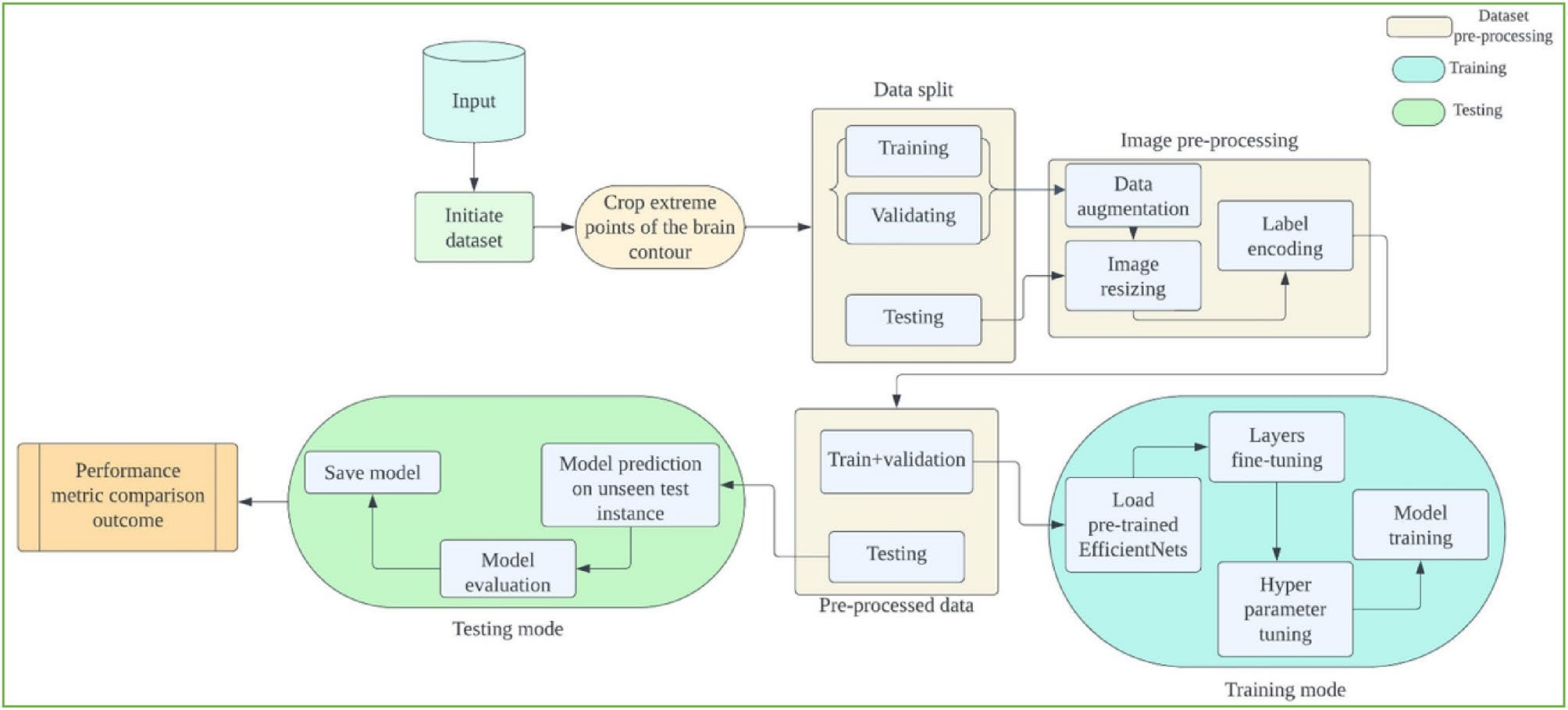
Proposed model block diagram.

In this section, we describe the methods used for categorizing different types of brain tumors using Magnetic Resonance (MR) Images. We present a visual representation of the approach in Fig. 1 through a schematic block diagram. To initiate the process, we start with magnetic resonance (MR) images containing brain tumor slices, which undergo several preprocessing steps. These steps are crucial for adequately preparing the samples before commencing the training. Recognizing the limitation posed by the initial dataset size in effectively training a deep convolutional neural network (CNN) architecture, we employ data augmentation techniques on the samples within the training set. This augmentation process results in the generation of additional slices for each specific tumor category. The methodology employed in this study capitalizes on transfer learning. We leverage pre-trained EfficientNets and their variations. Specifically, we fine-tune five distinct versions of pre-trained EfficientNet models, namely EfficientNetB0 through EfficientNetB4. These models are used for feature extraction and classification, with a specialized focus on MRI sequences obtained from a CE-MRI brain tumor dataset. The selection of these models is based on their computational efficiency, as they demand minimal floating-point operations per second (FLOPS) during inference. Additionally, their remarkable top-1 and top-5 accuracies on the ImageNet dataset, when compared to other modern pre-trained deep convolutional neural network (CNN) architectures, significantly influenced their choice. The subsequent sections delve into the intricate details of each stage comprising the proposed technique. This comprehensive exploration aims to provide a nuanced understanding of the inner workings of the method.

### Materials

**Figure 2:**
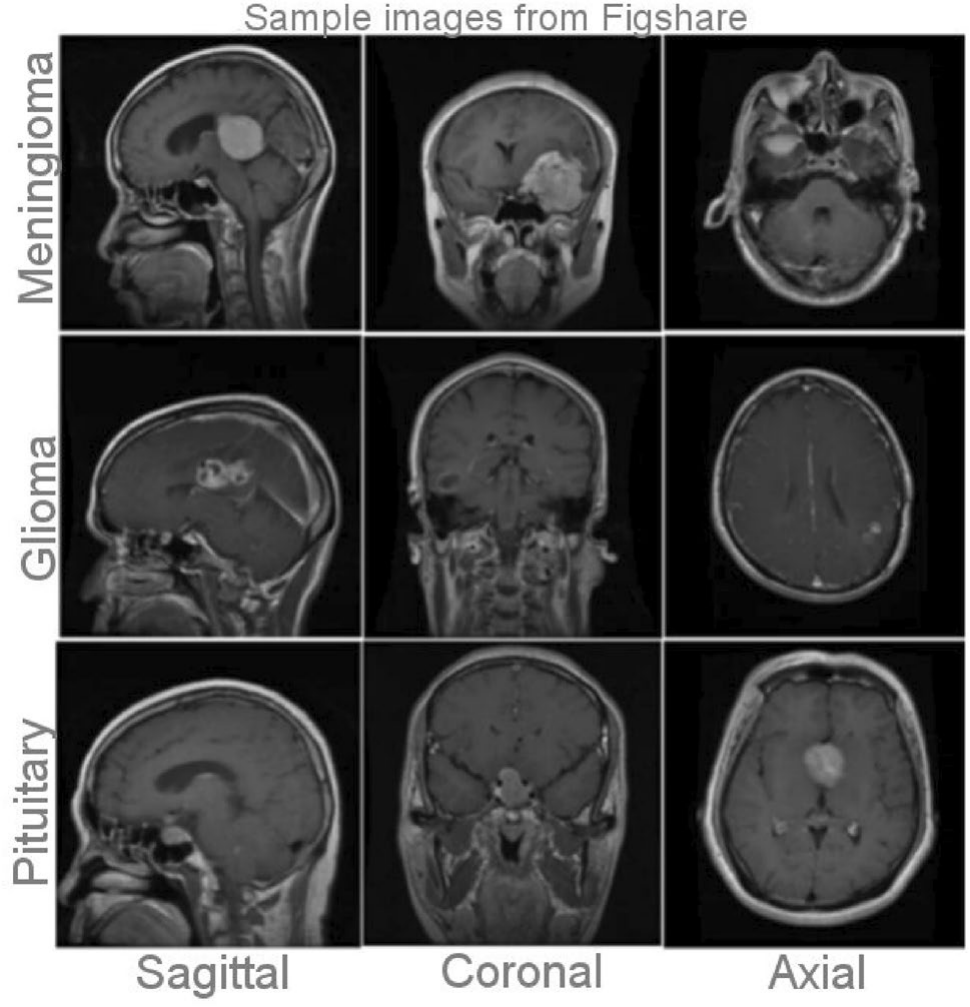
Sample dataset containing axial, coronal, and sagittal plane images of three different types of brain tumors: glioma, meningioma, and pituitary tumors.

The data used in this study is obtained from the publicly accessible Figshare brain tumor collection^30^. This collection, as depicted in Fig. 2, comprises a total of 3,064 T1-weighted Contrast-Enhanced Magnetic Resonance Images (CEMRI). These images encompass three distinct categories of brain tumors: glioma, meningioma, and pituitary tumor. The MR images are stored in MATLAB (.mat) format and have a resolution of 512 × 512 pixels. Each image entry contains essential information, including the class name, patient ID, image data, tumor borders (defined by x and y coordinates outlining various points on the tumor's boundary), and a binary tumor mask representing the segmented tumor area. The differentiation of brain tumor types and grades relies on factors such as their location, appearance, and dimensions^11^. To conduct the tests, three image planes are employed: axial, coronal, and sagittal. Table 1 provides an overview of the initial number of tumor instances for each tumor type.

**Table 1:** Distribution of data within each class following the division of the dataset into training and testing sets using 70:30 ratio.

Tumor type	Original: slice distribution	Data split-up	Total no. of slices in training set	Total no. of slices in testing set
Glioma	1426	70:30	998	428
Meningioma	708		496	213
Pituitary	930		651	280
Total	3064		2145	921

### Methods

A comprehensive explanation of each specific step within the suggested methodology is presented in the subsequent sections.

#### Pre-processing

To begin, the input images are down-sampled from 512 × 512 × 3 to 240 × 240 × 3 so that the resulting tensor can be properly processed by the pre-trained EfficientNet models. By maintaining image content and features during scaling, computational effort is reduced during network training.

**Figure 3:**
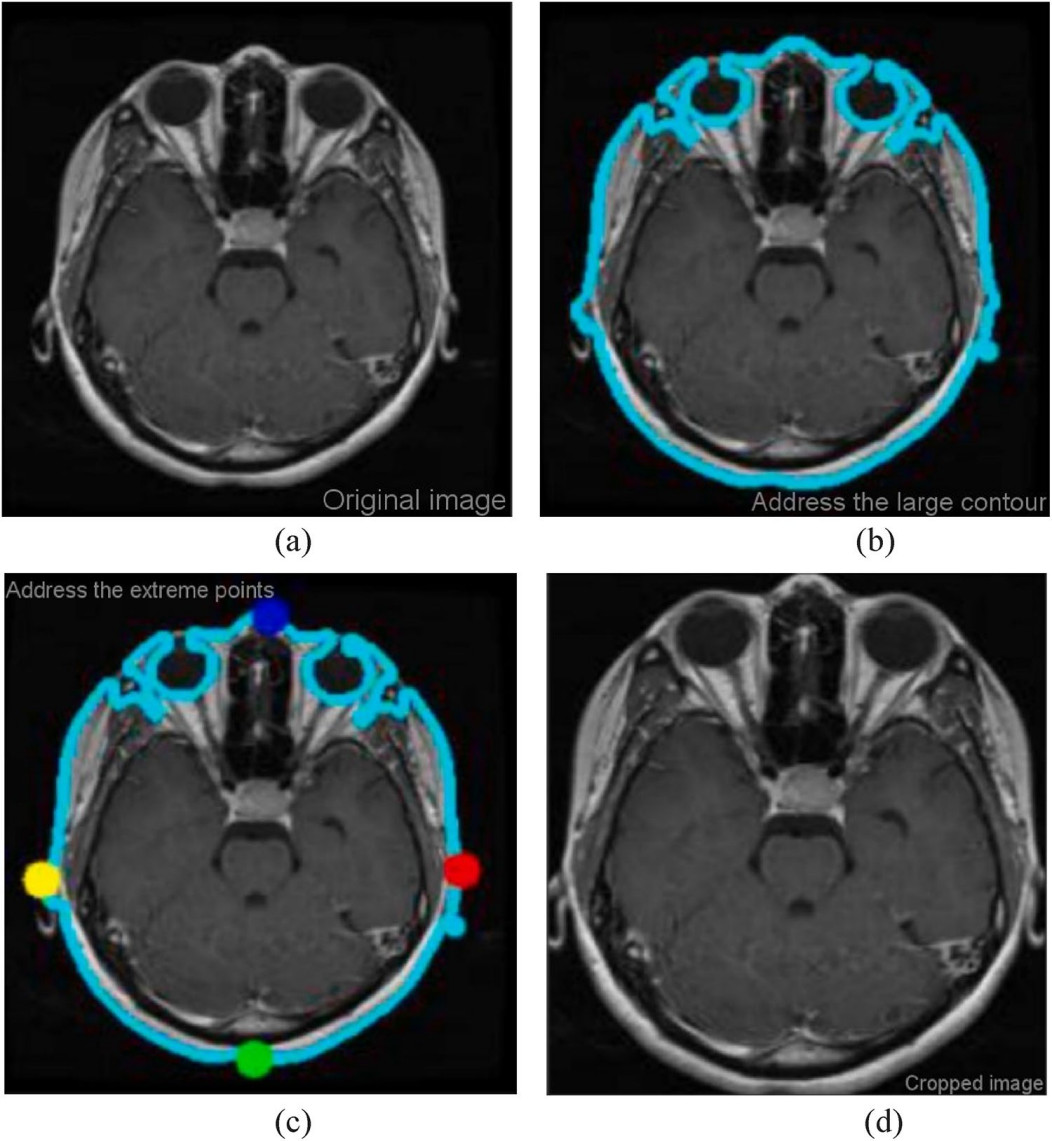
A series of steps is implemented to crop out unwanted regions MRI scans.

Figure 3 illustrates the process of extracting and eliminating the outer edges of the brain contour to crop the MR images. This involves the application of various image processing techniques to identify outliers. The sequence of operations begins with a grayscale conversion and the use of a Gaussian blur filter. Subsequently, thresholding is applied, and a series of erosion and dilation operations are carried out to further reduce noise. The thresholded image is then used to identify the largest contours after noise reduction. These contours determine the four corners of the original image: top, bottom, left, and right, effectively removing any extraneous image data that could contribute to noisy training. To ensure that the network doesn't focus solely on a specific subset of the dataset and to allow the system to train on unsorted images, data samples are randomly shuffled. After cropping the brain tumor MRI scans to remove irrelevant portions, the samples are divided in a 70:30 ratio between the training and test sets. Seventy percent of the data is allocated for training the model, while the remaining thirty percent is used to assess the model's performance on unseen test cases. Table 1 provides the class-wise distribution of the dataset after partitioning it into the training and test sets. After data augmentation, the class labels for glioma, meningioma, and pituitary tumors are encoded as 0, 1, and 2 in both the train and test sets, respectively.

#### Data augmentation

As the training set contains an inadequate number of samples to effectively train a deep CNN architecture, data augmentation is employed to address this limitation. Data augmentation is a widely-used technique that artificially increases the dataset size by applying various image manipulations, including rotation, scaling, flipping, mirroring, cropping, and more. By using this approach, a larger dataset can be generated without the need for additional data collection. In this research, we employ a variety of data augmentation techniques, primarily involving height shifts, to partially balance the instance distribution across each class in the dataset. This is achieved by increasing the height of glioma, meningioma, and pituitary tumor images by factors of 2, 5, and 4, respectively. Table 2 illustrates the distribution of images across classes within the training set before and after the application of data augmentation.

**Table 2:** Distribution of classes within the dataset prior to and post to the implementation of data augmentation on segments within the training set.

Tumor type	Tumor slices in a train set	
	Prior augmentation	Post augmentation
Glioma	998	2795
Meningioma	496	1290
Pituitary	651	1829
Total	2145	5914

#### Transfer learning

**Figure 4:**
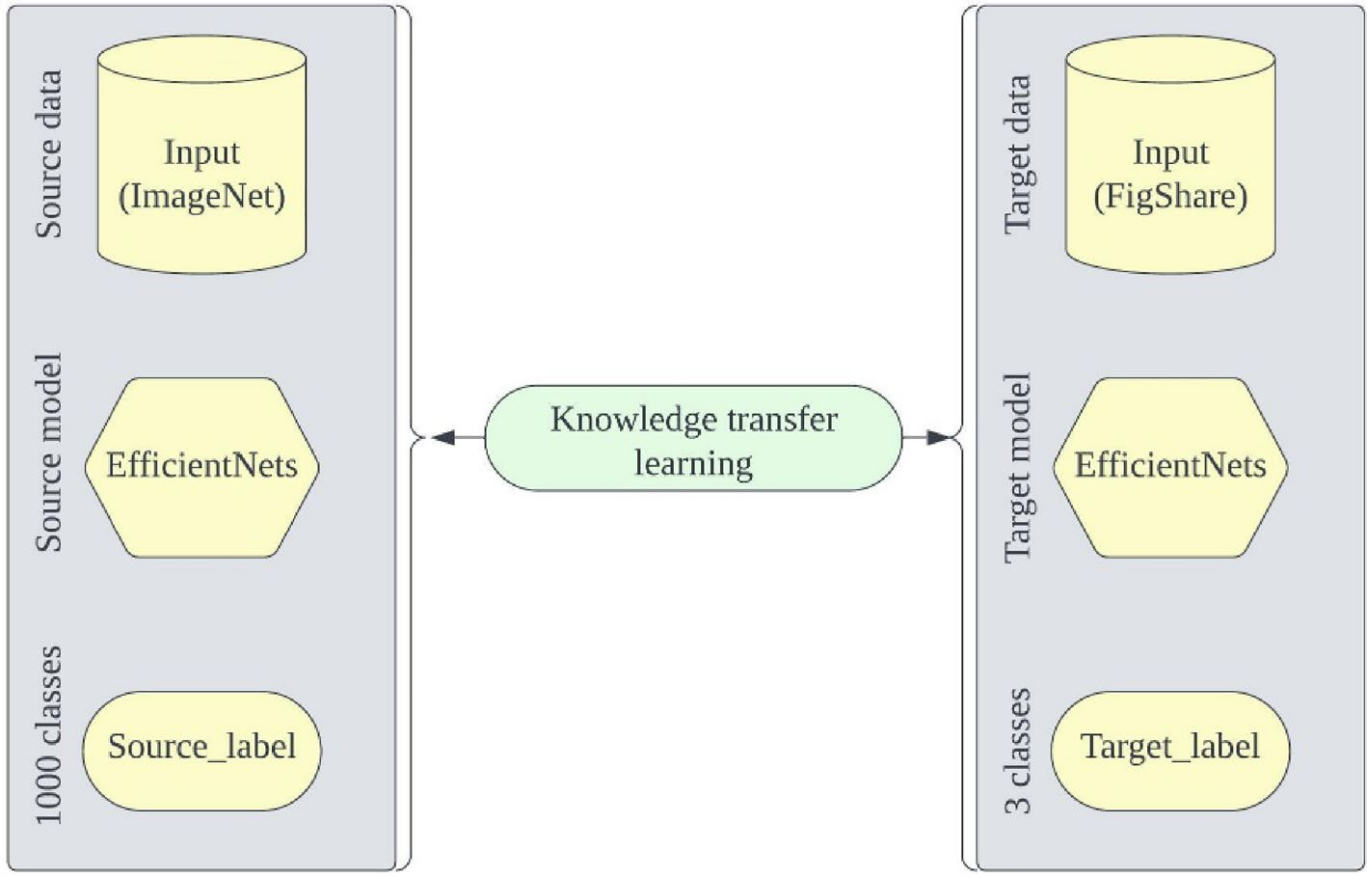
The fundamental concept of transfer learning.

In contrast to conventional machine learning methods, Convolutional Neural Networks (CNNs) have the advantage of automatically deriving both low-level and high-level feature maps from the model's convolutional foundation. Classification is performed by inputting these extracted feature maps into one or more densely connected layers, resulting in a one-dimensional feature vector. While CNNs have achieved significant success, they do have a drawback: they require a substantial dataset to train an accurate model and prevent overfitting. However, accumulating a large amount of annotated data is not always feasible, especially in research areas like medical imaging. Not only is a significant portion of the data unavailable, but access to it is also restricted. Transfer learning provides a solution to this challenge. It can address problems with limited data points, such as the classification of brain tumors from MRI scans. Transfer learning involves leveraging the knowledge acquired by architectures trained on extensive benchmark datasets like ImageNet and applying it to similar or even dissimilar problems. The broader concept of transfer learning is illustrated in Fig. 4. Given the disparity between the domains of the source dataset and the target dataset (in this case, MRI), it is unfeasible to directly use any pre-trained CNN architectures for inference and expect them to generalize well to unseen test instances. Instead, the layers of the pre-trained models need to be carefully fine-tuned to align with the specific characteristics of the images in the target domain. Fine-tuning involves the process of retraining the weights of the upper layers in a deep CNN architecture that was originally trained on an extensive dataset for specific tasks. Achieving the fine-tuning of pre-trained architectures can be done by either unfreezing some or all layers in the convolutional base or by using the pre-trained architectures as fixed feature extractors, which are then input into other classifiers, such as Support Vector Machines (SVM), for classification^31^. In this study, we employ transfer learning using a pre-trained EfficientNet model and fine-tune five distinct pre-trained EfficientNet models labeled as EfficientNetB0 through EfficientNetB4. These models are fine-tuned using MRI sequences from the CE-MRI brain tumor dataset. Subsequent sections will provide details on the criteria used for fine-tuning the classification layer of the pre-trained EfficientNet architectures, the experimental configurations for training and evaluating the model, and the model's performance on previously unseen test instances.

#### Fine-tuning pre-trained EfficientNets and their different versions

**Figure 5:**
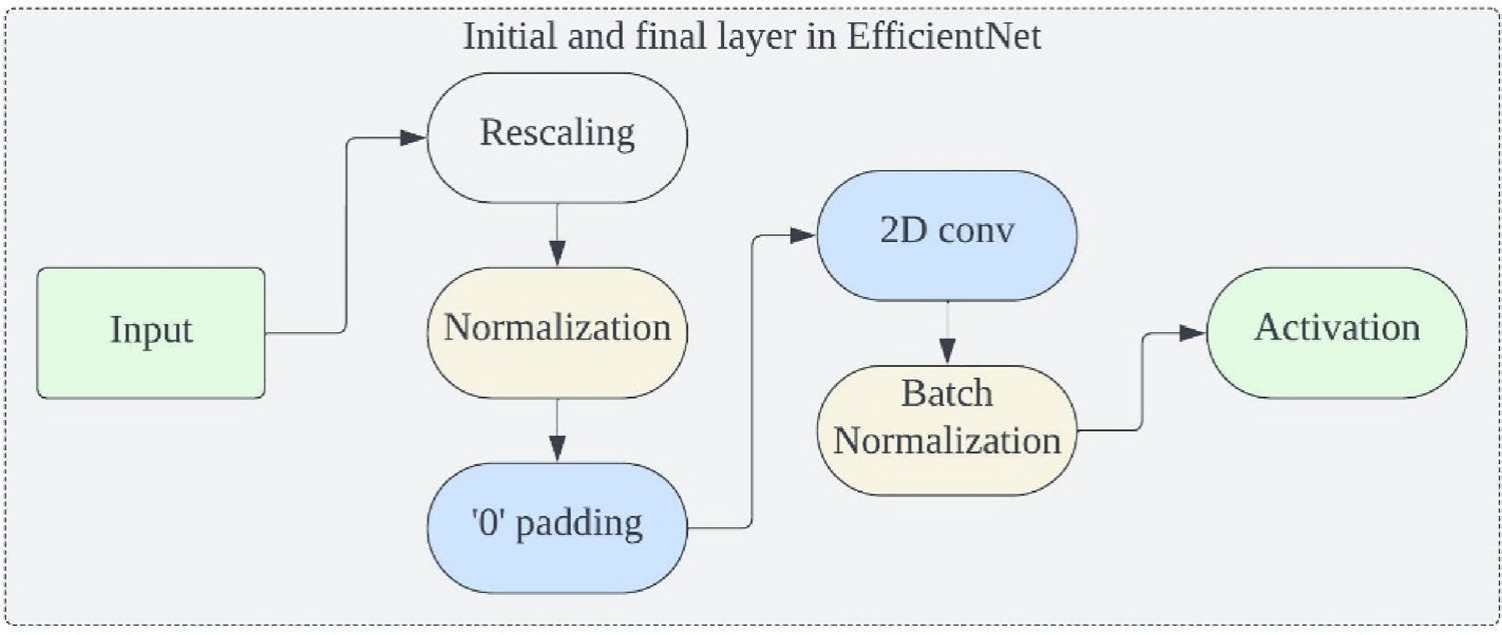
Initial and final block in EfficientNet.

**Figure 6:**
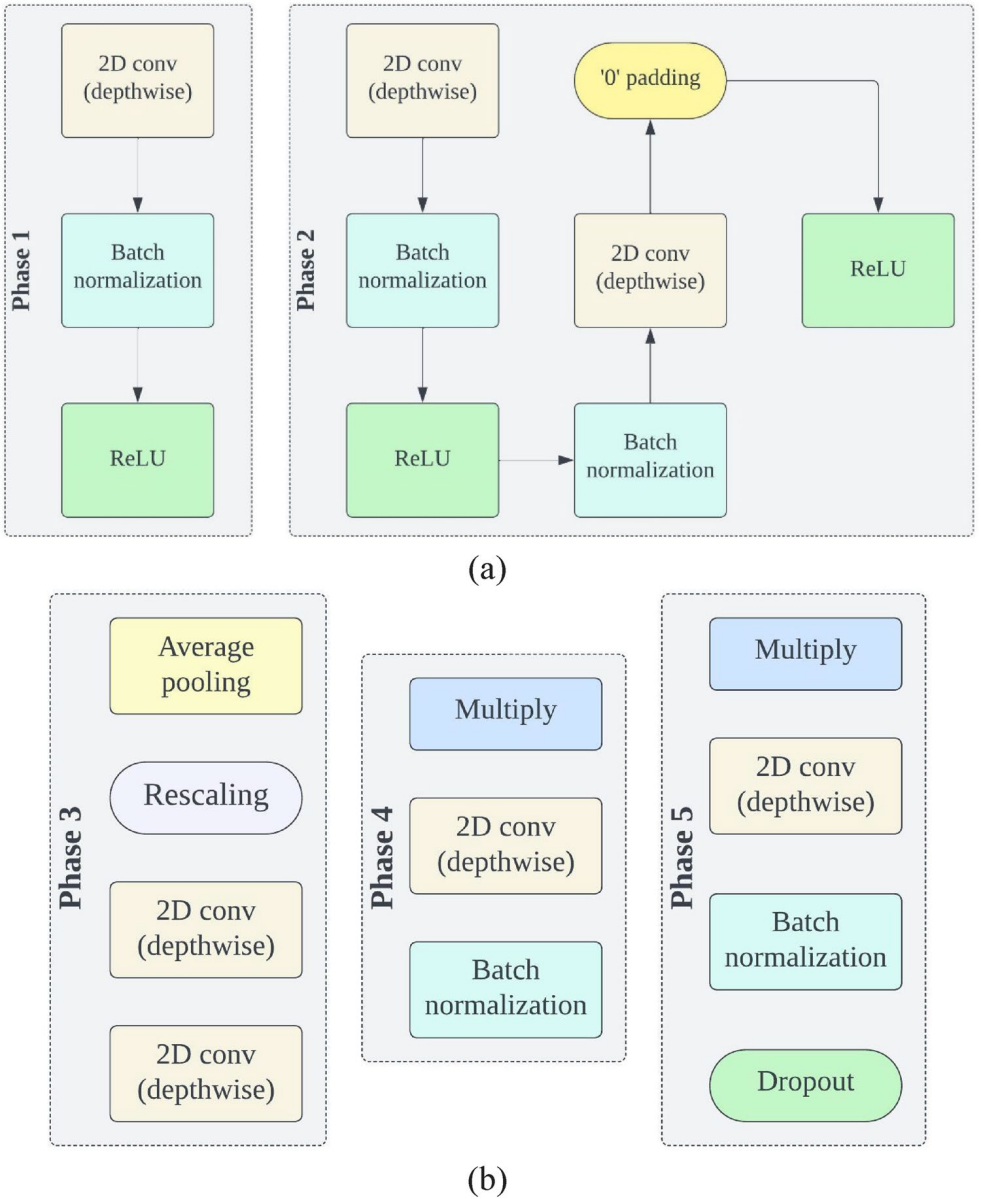
Five different phases of EfficientNets.

EfficientNets have demonstrated superior performance not only in ImageNet image classification but also in a range of transfer learning scenarios. Unlike traditional scaling methods used in previous studies, which involved arbitrary adjustments to the width, height/depth, and resolution of deep CNN architectures to improve their ability to generalize, EfficientNets adopt a consistent and systematic compound scaling strategy. This strategy systematically enhances the CNN architecture by using predefined sets of scaling coefficients. With these considerations in mind, researchers have introduced a series of foundational EfficientNet models, labelled as EfficientNetB0 through EfficientNetB7, which are suitable for both classification and segmentation tasks. The basic structure of all EfficientNets consists of an initial block, followed by seven additional blocks, and culminates in a final layer. This architecture is visually represented in Figs. 5 and 6.

**Figure 7:**
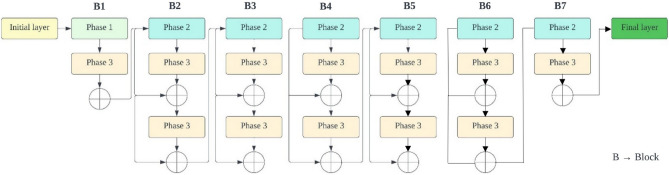
Architecture of EfficientNetB1–B2 (seven blocks).

Figure 7 illustrates the gradual increase in the number of modules across all blocks from EfficientNetB0 to EfficientNetB7. Each variant of the EfficientNet family has its unique range and configurations. For instance, the simplest version, EfficientNetB0, consists of 237 layers and 5.3 million parameters, while the most complex, EfficientNetB7, boasts 813 layers and 66 million parameters. EfficientNets also feature mobile inverted bottleneck convolution (MBConv) layers in their design, a concept similar to those found in MobileNetV2 and MnasNet. These networks are capable of handling image normalization automatically, accommodating input images with pixel intensity levels ranging from 0 to 255. In this study, we employ five distinct variations of EfficientNets, labelled as B0 through B4, as the foundation for categorizing different types of brain tumors. The choice of which variant to use depends on several factors, including dataset size, available resources for model training and evaluation, model depth, network parameters, and batch size. The larger variants of EfficientNets, such as EfficientNetB5 through EfficientNetB7, have more extensive network depth and parameters. This, in turn, increases the risk of overfitting the training data and demands higher computing resources (GPU + RAM) for training. Therefore, for multi-class brain tumor type classification tasks in this study, we exclusively utilize EfficientNetB0 to EfficientNetB4 as the backbone.

**Figure 8:**
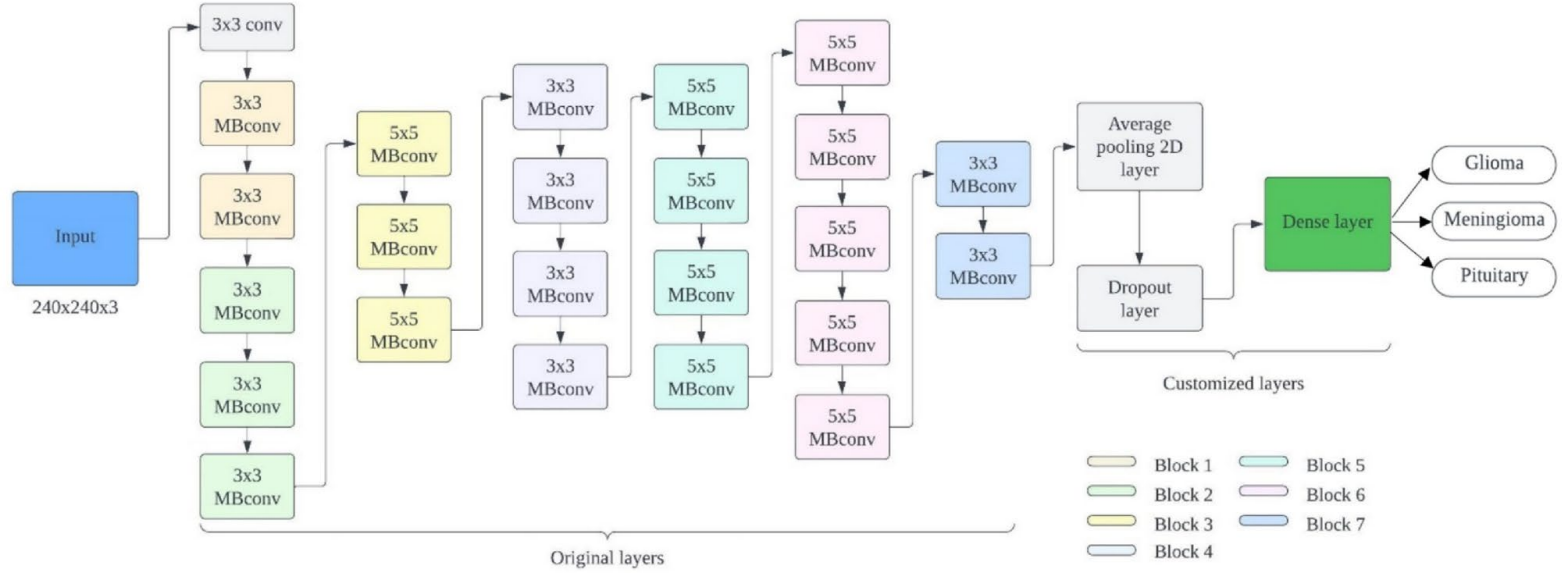
Proposed approach for refining the pre-trained EfficientNetB2 model.

In this study, we perform transfer learning on five different versions of pre-trained EfficientNets, which were originally trained using the ImageNet benchmark dataset. The considered versions encompass EfficientNetB0 through EfficientNetB4. These models undergo fine-tuning by adjusting them to work with MRI sequences extracted from the CE-MRI brain tumor dataset. Figure 8 illustrates the network architecture for the enhanced version of EfficientNetB2. The fine-tuning process of these pre-trained EfficientNets begins by initializing the base model with pre-trained ImageNet weights, providing a foundational structure for further adjustments. To reduce dimensionality, a Global Average Pooling (GAP) layer is introduced on top of the EfficientNets backbone, while the convolutional base of all EfficientNet blocks remains unchanged. This GAP layer also simplifies the network in terms of parameters without compromising model accuracy. After the GAP layer, a dropout layer with a probability of 0.5 is incorporated into the network. This dropout layer acts as a regularizer, preventing the model from becoming overly deterministic. Finally, the output layer, originally designed with 1000 units, is modified to contain three units, each followed by a softmax activation layer. This modification allows for the classification of three distinct brain tumor types. The entire model is then retrained using CE-MRI data from brain tumors.

## Experimental setup

In the beginning of this section, we will outline the necessary hardware and software requirements for training and evaluating the model. Subsequently, we will delve into the analysis of various hyperparameters, making explicit adjustments until an optimal combination is found. Finally, we will provide a concise explanation of each performance metric used during the model evaluation to conclude this section.

### System requirements

The experiments were conducted on Google Colaboratory, an open-source notebook platform provided by Google. This platform offers access to both free and premium GPU and TPU resources, which are valuable for academic and research purposes. The models were trained using four GPUs, each with 16 GB of RAM. The coding was done in Python, and the TensorFlow and Keras APIs were used for the backend and frontend of the system, respectively.

### Hyper parameter settings

Empirical fine-tuning of hyperparameters, which are training parameters, was conducted to find the most suitable settings for model training. These hyperparameters include batch size, optimizers, learning rate, epochs, and loss function. Categorical cross-entropy was chosen as the loss function since the task involves classifying brain tumors into glioma, meningioma, and pituitary tumor categories, constituting a multi-class classification challenge. The initial configuration for all five EfficientNets models involved using Adam as the optimizer and a learning rate of 0.001 (1e−2). To monitor the model's performance, the validation accuracy was observed every 5 iterations, and the initial learning rate was reduced by a decay factor of 0.3. A dropout rate of 0.2 was employed for further regularization during fine-tuning, without affecting the ImageNet weights. A mini-batch size of 32 images was used for the entire training dataset, and the fine-tuning of EfficientNets was performed over 50 epochs. To evaluate the model's performance and detect overfitting, 10% of the images from the training dataset were set aside as a validation set after each epoch. All four variants of EfficientNet (B0–B4) underwent training and evaluation using identical data and hyperparameters. The hyperparameters used throughout the study are summarized in Table 3, along with their respective optimal values.

**Table 3:** Hyper-parameter list and corresponding values.

Hyper parameters	Ranges
Shape of input	240 × 240 × 3
Connect rate of drop layer	0.2
Activation function of output layer	Softmax
No. of epochs	50
Batch_size	32
Optimizer	Adam
Learning rate (initial)	0.001
Decay factor rate of learning	0.3
Patience level	5
Validation split	0.1
Function of loss	Cross entropy

### Performance metric evaluation

Accuracy, precision, recall, sensitivity, specificity, and F1-score are among the performance metrics employed to assess the overall performance of the proposed model. Furthermore, the generation of a confusion matrix highlights the class-wise predictions made by the model on unseen test examples. Subsequent subsections will delve deeper into each evaluation metric, offering more comprehensive discussions following a brief description of each.

#### Sensitivity (Se)

In the context of medical diagnosis, particularly in tasks like brain tumor categorization, the model’s sensitivity and recall have a crucial role in determining the presence or absence of a brain tumor in a patient. Sensitivity, also known as the true positive rate (TPR), is pivotal; it represents the proportion of accurately predicted positive labels that are indeed positive. The following formula enables the quantification of the model’s sensitivity and recall:

1 $$Se=\frac{TP}{TP+FN}$$

#### Specificity (Sp)

Specificity, also known as the true negative rate (TNR), signifies the percentage of predicted negative labels that are indeed negative. The formula for computing specificity is presented below in the form of an equation,

2 $$Sp=TNR=\frac{TN}{TN+FP}$$

#### Accuracy (Acc)

The accuracy of the model is determined by the ratio of correctly predicted labels to the total number of labels. This yields a percentage that reflects the expected accuracy of the tested model. A formula is available for computing precision, which is a key evaluation metric in classification tasks.

3 $$Acc=\frac{TP}{TP+TN+FP+FN}$$

#### F1-score

The F1-score, commonly referred to as the F-measure, serves as the harmonic mean between a model’s accuracy and recall. This metric offers a comprehensive assessment of the model’s overall performance. The F1-score underscores the need for a balance between precision and recall. The formula for calculating the F1 score is presented below,

4 $$F1 score=\frac{2\times Pr\times Recall}{Pr+Recall}$$

#### Confusion matrix

A confusion matrix, sometimes referred to as an error matrix, is a structured table used to display data about actual labels (ground truth) and predicted class assignments. It not only provides an overall summary of the model's performance but also offers a more detailed insight into how well the model generalizes across individual classes. The layout of the confusion matrix typically positions the ground truth along the y-axis, while the predicted class labels are represented along the x-axis.

## Experimental results and discussion

**Figure 9:**
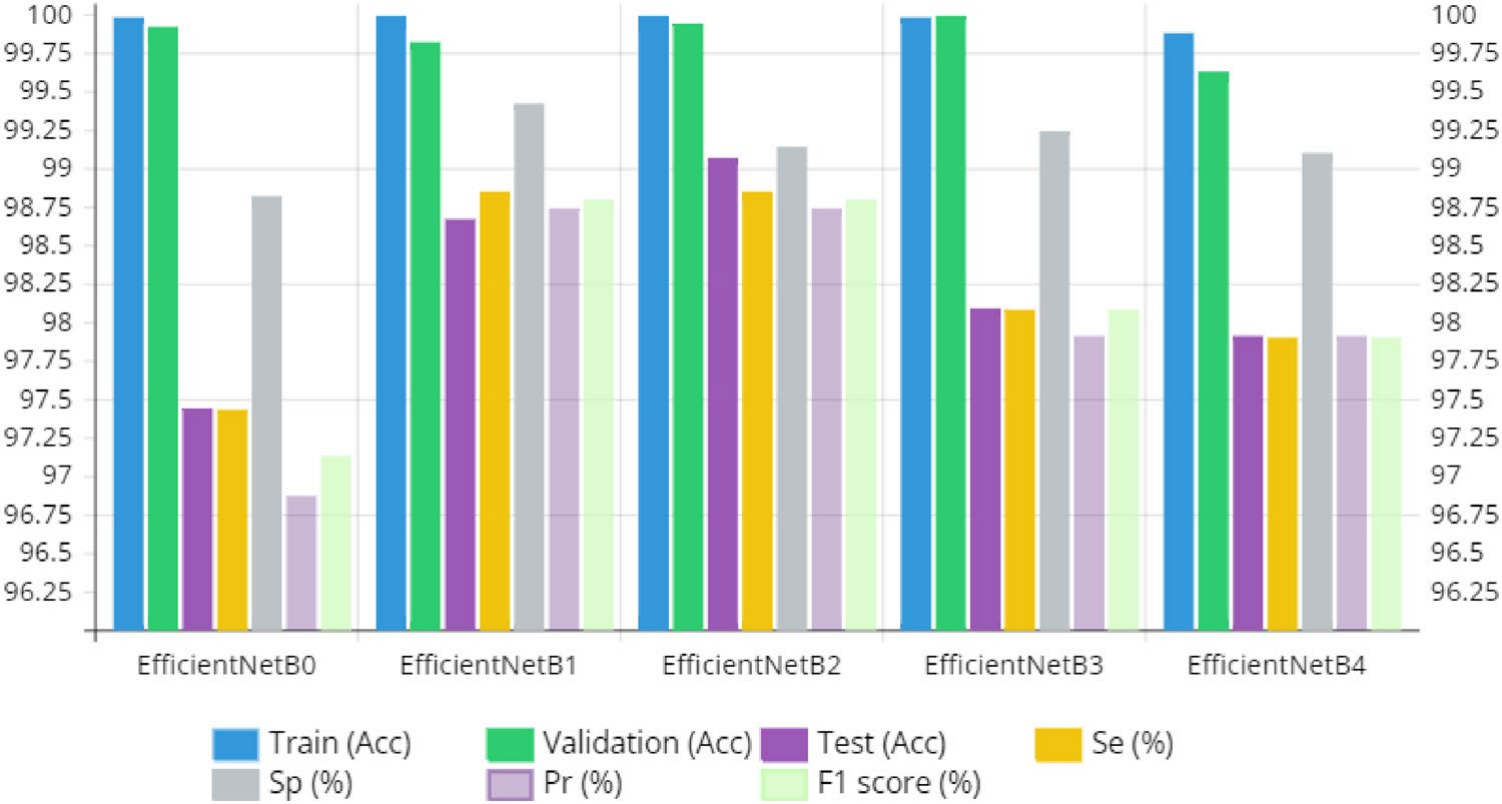
Training and validation outcomes of the fine-tuned EfficientNets B0–B4.

**Figure 10:**
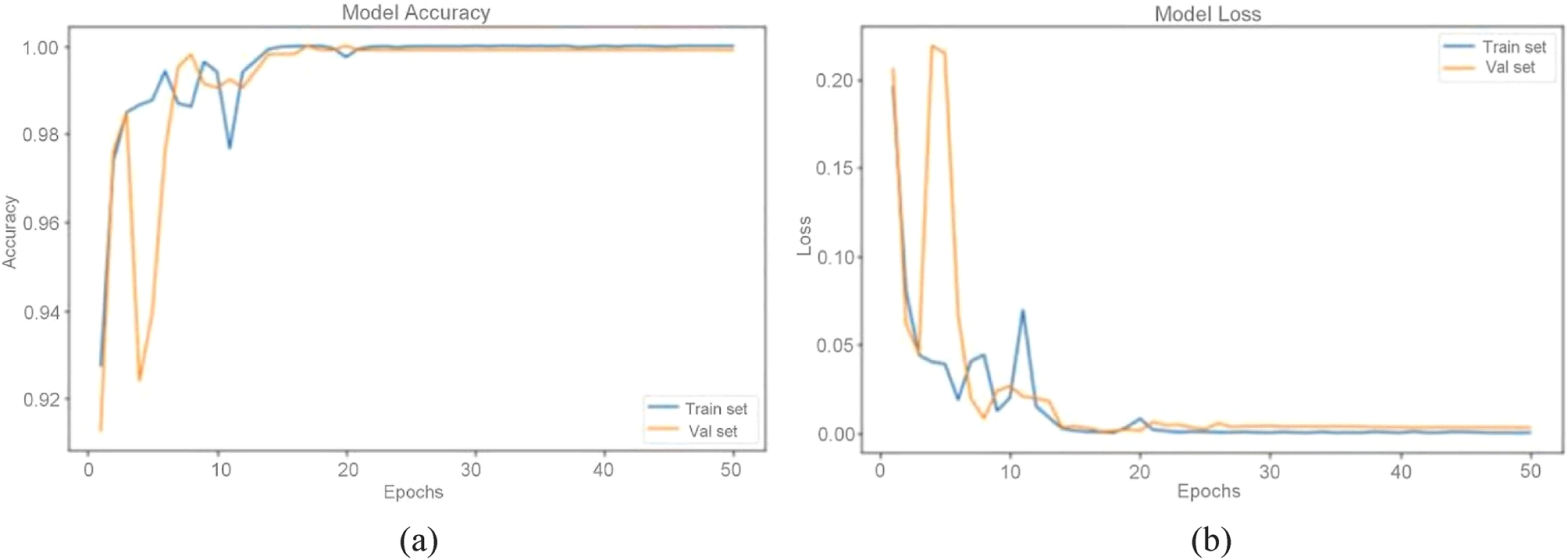
Proposed fine-tuned EfficientNetB2 model's (**a**) training, (**b**) test accuracy-loss curves.

In this section, we present the results of the analysis conducted on the trained and fine-tuned variations of EfficientNets for the multi-classification of brain tumor types, which include glioma, meningioma, and pituitary tumors. These results were derived from testing the performance of the EfficientNets on unseen data. Table 4 provides a summary of the predictions made by the fine-tuned EfficientNetB0 to B4, presenting metrics such as precision, recall, F1-score, sensitivity, and specificity for each category. The suggested approach, fine-tuning the pre-trained EfficientNetB2, demonstrated robust performance across all evaluation criteria. Among the classes, glioma achieved the highest test accuracy of 99.60%, while pituitary tumor closely followed with a test accuracy of 99.58%. In contrast, meningioma exhibited the least accurate classification with an accuracy of 97.88%. This discrepancy can be attributed to the relatively smaller number of test samples available for meningioma compared to the other classes, as indicated in Table 1. Figure 9 depicts the findings from the training and validation of the fine-tuned EfficientNets B0–B4. EfficientNetB2 outperformed the other variations significantly, achieving an F1-score of 98.71%, a test accuracy of 98.86%, precision of 98.65%, and recall of 98.77%. In comparison, EfficientNetB0 faced challenges related to generalizability, with a test accuracy of 97.39%. This can be attributed to its lower complexity as the least complex member of the EfficientNets family, containing only 237 layers. The accuracy and loss curves for both model training and validation are illustrated in Fig. 10a,b. These curves show that the model's accuracy consistently increased during training and validation, while the loss gradually decreased. Notably, there is a substantial rise in validation accuracy and loss between the 5th and 10th epochs. After this point, the training process exhibited smoother progress with each successive epoch.

**Table 4:** The proposed fine-tuned EfficientNet B0-B4 class-wise assessment on test data.

Model	Tumor type	Se (%)	Sp (%)	Acc (%)	Pr (%)	F1 score (%)
EfficientNetB0	Glioma	97.27	99.42	97.28	99.36	98.30
	Meningioma	96.54	97.92	96.55	93.27	94.88
	Pituitary	98.45	99.09	98.46	97.94	98.19
EfficientNetB1	Glioma	99.02	99.43	99.03	99.38	99.20
	Meningioma	97.96	98.99	97.96	97.28	97.62
	Pituitary	99.53	99.81	99.00	99.54	99.54
EfficientNetB2	Glioma	99.02	99.13	99.68	99.38	99.20
	Meningioma	97.96	98.99	97.96	97.28	97.62
	Pituitary	99.53	99.26	99.54	99.54	99.54
EfficientNetB3	Glioma	98.67	99.73	98.68	99.72	99.19
	Meningioma	96.54	98.98	96.55	96.55	96.55
	Pituitary	98.99	98.97	99.00	97.43	98.46
EfficientNetB4	Glioma	98.67	99.42	98.68	99.72	99.19
	Meningioma	96.54	98.98	96.55	96.55	96.55
	Pituitary	98.45	98.87	98.46	97.42	97.93

**Figure 11:**
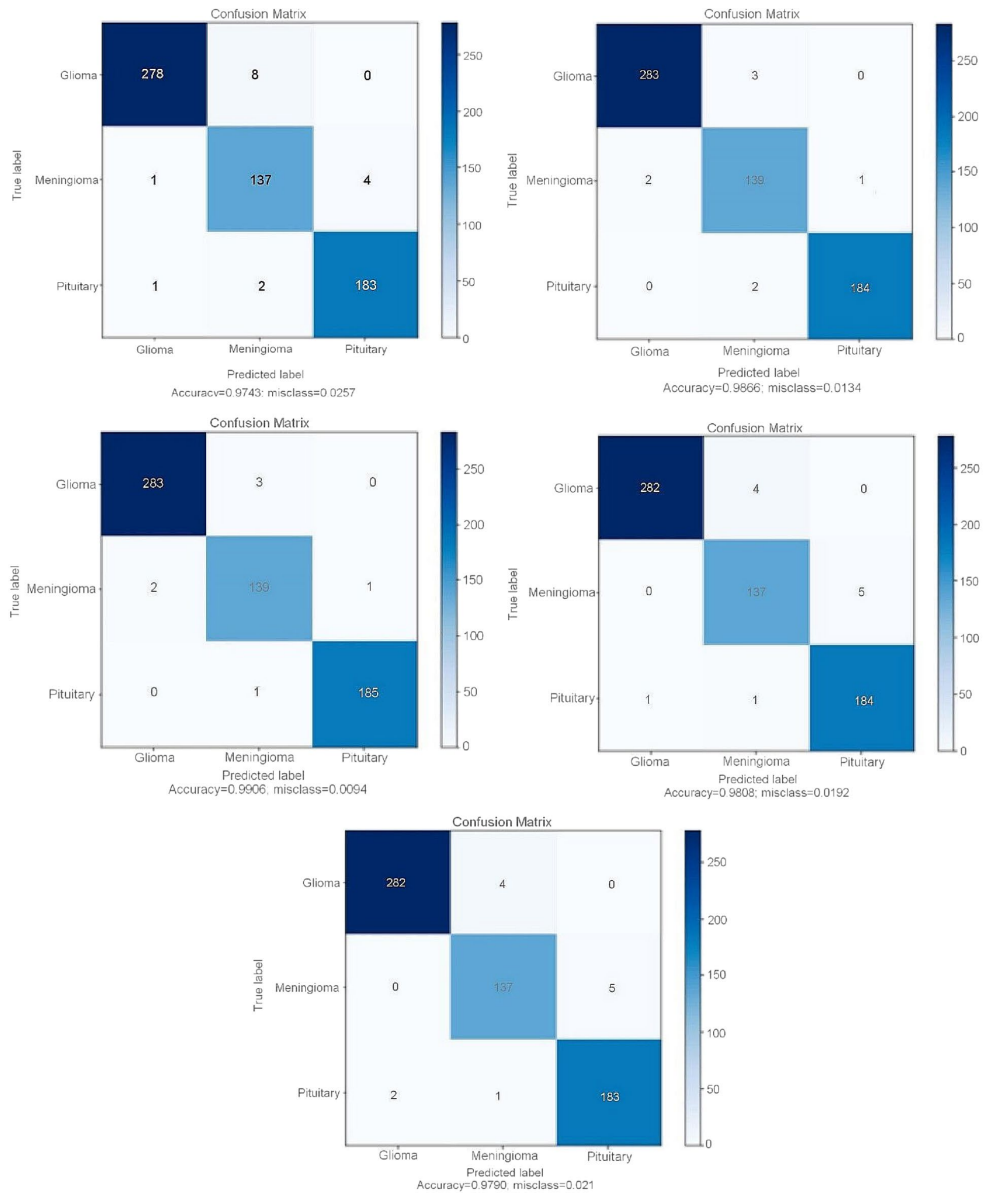
Confusion matrix of the proposed EfficientNets B0–B4.

Figure 9 provides a histogram that illustrates the comparison of performance among the fine-tuned EfficientNet B0–B4 models in terms of accuracy, precision, sensitivity, specificity, and F1-score. This histogram allows for a clear visual representation of the models' performance across these key metrics. You can refer to the histogram for a detailed overview of how the models compare in terms of these evaluation criteria. Figure 10a,b depicts the proposed EfficientNetB2 model's training and test accuracy-loss curves. The comprehensive performance of the fine-tuned EfficientNet B0–B4 models in predicting and evaluating the test data is further depicted through a confusion matrix, as shown in Fig. 11. In the context of the confusion matrix, meningioma stands out as the class with the highest False Negative Rate (FNR), which contributes to its relatively lower accuracy, as evident from the information provided in the confusion matrix. Conversely, pituitary tumors exhibit the highest accuracy, recall (sensitivity), F1-score, and specificity, with values of 99.54%, 99.53%, 99.54%, and 99.81%, respectively.

### Impact of data augmentation on the proposed model's ability to generalize

The improvement in the model's performance in terms of accuracy, speed (during training and inference), and model size was achieved through careful selection of the network architecture and the implementation of data augmentation. To evaluate the impact of data augmentation on model training and evaluation, a series of experiments was conducted. Both pre-trained EfficientNetB1 and EfficientNetB2 models were trained and evaluated in two different scenarios: one with data augmentation and the other without it. The results of these experiments are summarized in Table 5, highlighting the positive effect of data augmentation on the model's ability to generalize to previously unseen test cases. The decision to compare EfficientNet B1 and B2 was based on several considerations. EfficientNet models are known for their trade-off between model size and performance, with B1 being smaller and computationally less expensive, while B2 is slightly larger and potentially offers improved performance. This trade-off is representative of the EfficientNet family, and the goal was to assess how this trade-off impacts the model's performance in the context of the study. The choice of model often depends on the available computational resources and deployment environment, so comparing B1 and B2 aimed to provide insights for practitioners with various hardware and latency requirements. Specifically, the accuracy improved from 98.41 to 98.66% for EfficientNetB1 and from 98.57% to 99.06% for EfficientNetB2. During these experiments, several key observations were made.

**Table 5:** Evaluation of fine-tuned EfficientNet B1-B2 performance prior and post data augmentation.

Model	Accuracy of test	
	Prior augmentation	Post augmentation
EfficientNetB1	98.41%	98.66%
EfficientNetB2	98.57%	99.06%

### Proposed model's computational complexity

As part of this research, an investigation was conducted to assess the model's robustness, including its speed during both training and inference, total parameters, and overall size. The performance of the proposed and fine-tuned EfficientNet models, including B0 through B4, was compared, taking into account the previously discussed metrics. Each of these models offers a different balance between model size, speed, and accuracy. EfficientNetB0, having the fewest parameters, is the fastest and most lightweight option. However, this trade-off results in a slight reduction in model accuracy. Both EfficientNetB1 and EfficientNetB2 are reliable models with limited parameter counts and compact representation sizes. EfficientNetB2, in particular, stands out as it achieves a balance between accuracy and robustness. The summary of these comparisons can be found in Table 6.

**Table 6:** Fine-tuned EfficientNet B0-B4 training, inference, #parameters, and model size comparison with Xception, Inception-ResNetV2, and NasNet-Large.

Model	Training duration (m)	Inference time (s)	Parameters (million)	Size (MB)	Test accuracy (%)
ResNetv2	79	10	56.7	201	98.05
Xception	76	10	23.71	611.1	97.81
NasNet-Large	99	24	85.1	727.1	97.1
EfficientNetB0	91	2	4.1	48	97.43
EfficientNetB1	128	2	6.61	77.1	98.66
EfficientNetB2	133	2	7.81	89.9	99.06
EfficientNetB3	169	2	10.83	125	98.08
EfficientNetB4	221	3	17.85	204.1	97.9

### Presenting grad-CAM visualization for the proposed EfficientNetB2 using previously unexamined test data

Grad-CAM offers a visual representation of how EfficientNetB2 makes predictions when classifying different types of brain tumors from MRI sequences. It operates by utilizing the gradient of a specific target concept, which is fed into the final convolutional and activation layer. This process generates a coarse localization heatmap, highlighting the relevant area in the original image crucial for predicting the target concept. In essence, it reveals the significance of specific regions in the original image. The middle row of images displays EfficientNetB2's predictions for each tumor type, based on data it has not encountered previously. In contrast, the top row showcases attention maps derived from the model's final activation layer. These attention maps illustrate the areas the model focuses on when making predictions. In the last step, a Grad-CAM heatmap is created by applying the attention map from the feature space to the original image. This heatmap effectively emphasizes the tumor regions within the MRI image for each tumor category.

### External cohort cross-dataset validation

The proposed technique was subjected to cross-dataset validation using an external dataset to further demonstrate its robustness. For this purpose, a brain tumor classification dataset was obtained from the open-source Kaggle repository and utilized. This dataset is divided into train and test folders, with MRI slices belonging to one of four classes: normal, glioma, meningioma, and pituitary tumor, as shown in Table 7. The proposed model was not trained on normal instances, so the normal class was excluded from the dataset for cross-validation. Both train and test MRI images were used for cross-validation on this external cohort. Table 8 provides a summary of the results obtained from the cross-dataset validation on the external cohort. Among the various fine-tuned EfficientNet models, EfficientNetB2 achieved the highest performance. It demonstrated precision, recall/sensitivity, specificity, F1-score, and accuracy values of 92.11%, 92.11%, 95.96%, 92.02%, and 92.23%, respectively. These results highlight the significant resilience of the suggested modifications to EfficientNets when applied to an external dataset.

**Table 7:** Brain tumor classification dataset class distribution in train and test sets.

Split	Total no. of instance	Glioma	Meningioma	Pituitary	Benign
Training	2870	831	819	829	391
Testing	394	102	117	80	95

**Table 8:** Cross-dataset validation of fine-tuned EfficientNets on external correlations.

Model	Acc	Se (%)	Sp (%)	Pr (%)	F1 score (%)
EfficientNetB0	86.92	86.98	92.96	87.44	86.91
EfficientNetB1	90.67	90.21	94.81	90.31	90.02
EfficientNetB2	92.23	92.11	95.96	92.11	92.02
EfficientNetB3	86.10	86.20	92.76	87.73	85.98
EfficientNetB4	87.76	85.61	93.38	88.91	87.68

### Performance metric comparison of the proposed and state-of-the-art methods

In this section, we carry out a performance comparison between the proposed fine-tuned EfficientNetB2 and several state-of-the-art approaches developed from 2019 to 2023. Table 9 displays the results of this performance comparison, including metrics such as test accuracy, average precision, recall (sensitivity), and specificity. It's worth noting that while the proposed model demonstrates impressive performance, it also involves substantial computational costs in terms of network parameters, model size, and FLOPS (floating-point operations per second).

**Table 9:** Classification accuracy comparison of proposed and state-of-the-art methods.

Author	Year	Dataset	Method	Classification accuracy (%)
Ali Ari^27^	2018	DICOM	Extreme learning machine local receptive fields	97.18
Ali Mohammad Alqudah^22^	2019	Figshare	CNN	98.93
Arshia Rehman^23^	2019	Figshare	VGG16	98.69
Francisco Javier Díaz-Pernas^25^	2021	Nanfang Hospital, Guangzhou, China, and General Hospital	Deep convolutional neural network	97.3
Muhammad Aamir^28^	2022	Figshare	Partial least squares	98.95
Alok Sarkar^32^	2023	Kaggle	AlexNet CNN	98.15
Proposed model	2023	Figshare	Fine-tuned EfficientNetB2	99.06

In this study, we have ventured into uncharted territory by applying fine-tuning techniques to the EfficientNet architecture using the CE-MRI brain tumor dataset. Our primary goal was to develop a model capable of classifying brain tumors into one of three categories: glioma, meningioma, or pituitary tumor. While the use of EfficientNets in various domains is well-documented, its application in this particular context marks a novel and innovative approach. The results, thoughtfully presented in Table 9, underscore the remarkable success of our proposed method. Through the careful process of fine-tuning a pre-trained EfficientNetB2 model and integrating data augmentation, we have achieved a level of performance that surpasses existing state-of-the-art methods. These results provide clear evidence of the model's effectiveness in classifying brain tumors with unprecedented precision. Our model's superior performance is most apparent in the overall test accuracy, which reaches an impressive 99.06%. This measure reflects the model's capacity to correctly classify brain tumors into their respective categories with a remarkable degree of accuracy. Furthermore, precision, at 98.73%, highlights the model's ability to minimize false positives, ensuring that when it identifies a tumor, it is highly likely to be correct. The recall rate, standing at 99.13%, showcases the model's proficiency in minimizing false negatives, indicating its capacity to detect the vast majority of actual tumor cases. Finally, the F1-score, which harmonizes the trade-off between precision and recall, reaches an outstanding 98.79%, reinforcing the model's overall excellence. In summary, this study not only pioneers the application of EfficientNets in brain tumor classification but also significantly raises the bar in terms of performance and accuracy. Our findings underscore the potential of deep learning and transfer learning techniques in the field of medical imaging, particularly for the critical task of diagnosing brain tumors. This research opens new avenues for further exploration and promises to have a substantial impact on the medical community.

## Conclusion

This study utilizes transfer learning with five variations of pre-trained EfficientNets (EfficientNetB0 through EfficientNetB4) to perform multi-class classification of brain tumors using MR images of three tumor types: glioma, meningioma, and pituitary tumor. The pre-trained ImageNet weights are loaded into the foundational model, and the architecture of EfficientNet B0–B4 is adjusted by adding several top layers, including a convolutional base, a dropout layer, and a fully connected layer. For the multi-class classification of brain tumor types, a classifier is constructed on top of the pre-trained EfficientNets' convolutional base. The model is trained using the CE-MRI Figshare brain tumor dataset, with fine-tuning of the hyperparameters for EfficientNet B0–B4. The proposed fine-tuned EfficientNets are rigorously tested in multiple trials to evaluate their performance against other pre-trained models. The introduction of new data significantly improves the generalizability of the refined version of EfficientNetB2. Additionally, attention maps of features for the suggested fine-tuned EfficientNetB2 are visualized using Grad-CAM, effectively highlighting the areas of brain cell tumors. The scalability of the proposed approach is demonstrated through cross-dataset validation with an external cohort. The method involving the fine-tuning of pre-trained EfficientNetB2 as a backbone achieves impressive overall test accuracy, precision, recall, and F1-score metrics of 99.06%, 98.73%, 99.13%, and 98.79%, respectively. These results surpass several state-of-the-art methods that address similar classification challenges.

## Future work

In the context of future research, there is a promising avenue to explore the use of transformer-based architectures for brain tumor type classification. Departing from traditional deep CNN methods, this shift has the potential to offer a new perspective. The focus would be on extracting more comprehensive and information-rich feature maps, which could enhance the model's ability for pattern recognition. Moreover, the notion of partially simplifying network complexity introduces a dynamic element to this ongoing endeavour. This concept underscores the ever-evolving nature of research and opens the door to innovative advancements in brain tumor classification.

## Data Availability

The datasets used during the current study are available from the corresponding author on reasonable request.
